# Outcomes of intravenous and inhalation anesthesia on patients undergoing esophageal cancer surgery: a retrospective observational study

**DOI:** 10.1186/s12871-023-02023-1

**Published:** 2023-03-02

**Authors:** Yue Ma, Jie Ren, Zhuo Chen, Jingwen Chen, Ming Wei, Yu Wang, Hong Chen, Liping Wang

**Affiliations:** 1 Department of Anesthesiology, Harbin Medical University Cancer Hospital, No. 150 Haping Rd., Nangang District, Harbin, 150081 China; 2 Department of Anesthesiology, Guizhou Provincial People’s Hospital, No. 83 Zhongshan East Road, Nanming District, Guiyang, 550002 Guizhou China

**Keywords:** TIVA, INHA, Esophageal cancer, Overall survival, Disease-free survival

## Abstract

**Background:**

Different anesthetics may have opposite effects on the immune system, thus affecting the prognosis of tumor patients. Cell-mediated immunity forms the primary defense against the invasion of tumor cells, so manipulation of the immune system to produce an enhanced anti-tumor response could be utilized as an adjuvant oncological therapy. Sevoflurane has proinflammatory effects, while propofol, has anti-inflammatory and antioxidant effects. Therefore, we compared the overall survival (OS) and disease-free survival (DFS) of patients with esophageal cancer under total intravenous anesthesia and inhalation anesthesia.

**Methods:**

This study collected the electronic medical records of patients undergoing esophagectomy from January 1, 2014 to December 31, 2016. According to the intraoperative anesthetics, the patients were divided into total intravenous anesthesia (TIVA) group or inhalational anesthesia (INHA) group. Stabilized inverse probability of treatment weighting (SIPTW) was used to minimize differences. Kaplan–Meier survival curve was established to evaluate the correlation between different anesthesia methods in overall survival and disease-free survival of patients undergoing esophageal cancer surgery.

**Results:**

A total of 420 patients with elective esophageal cancer were collected, including 363 patients eligible for study (TIVA, *n* = 147, INHA, *n* = 216). After SIPTW there were no significant differences between two groups in overall survival and disease-free survival. However, the adjuvant therapy was statistically significant in improving OS, and the degree of differentiation was correlated with OS and DFS.

**Conclusions:**

In conclusion, there were no significant difference in overall survival and disease-free survival between total intravenous anesthesia and inhalational anesthesia in patients undergoing esophageal cancer surgery.

## Synopsis

Different anesthesia modalities may have different effects on tumor outcomes, we aimed to investigate the effect of intravenous anesthesia or inhalational anesthesia on the long-term prognosis in esophageal cancer in this work.

## Background

Esophageal cancer (EC) is a worrying health threat in China, ranking sixth among new cancer cases in 2020, with approximately 320,000 new claims and the 4th highest mortality rate. The incidence of EC in China is much higher than that in Western countries, with more than half of new annual cases of EC worldwide occurring in China [1]. EC is one of the most fatal malignancies with very poor overall 5-year survival rates (10% ~ 40%) [2]. Esophageal squamous cell carcinoma (ESCC) is the most common pathological type of EC in China. In contrast, esophageal adenocarcinoma is the predominant type in the Western World [3, 4]. The mainstream treatment method is radical resection of esophageal cancer [5].

Anesthetic techniques have varying effects on innate and cellular immunity, activation of adrenergic inflammatory pathways, and activation of cancer promoting cellular signaling pathways; these effects may translate into an influence of anesthetic technique on long term cancer outcomes [6, 7]. Laboratory and animal studies have suggested that volatile anesthetic drugs are more likely to enhance the activity of the cancer cells through suppression of immune cell function, modulation of the neuroendocrine stress response to surgery, and cancer cell signaling [8]. Other study also shown that volatile anesthetics used in cancer surgery may be associated with worse tumor prognosis [9]. In contrast, intravenous anesthetic agents such as propofol have anti-inflammatory and anti-oxidative effects that may protect against perioperative immune suppression [8].

At present, there is no conclusive evidence to support the superiority inhalational or intravenous anesthesia in esophageal cancer surgery. Therefore, we conducted a retrospective study to compare the overall survival and disease-free survival of patients with esophageal cancer surgery using Propofol-based TIVA and sevoflurane-based INHA.

## Methods

### Patient identification and exclusion

Electronic medical records of all patients who underwent elective surgery esophageal cancer at our hospital from January 1, 2014 to December 31, 2016 were collected. The exclusion criteria were as follows: (1) Patients with esophageal tumors except squamous cell carcinoma or adenocarcinoma. (2) Emergency surgery. (3) Patients with metastasis. (4) Two forms of anesthesia were received during the surgery. (5) Patients with incomplete clinical data and were lost follow-up. Patients who underwent surgery for esophageal cancer and had postoperative pathology were included.

### Anesthesia technique and grouping method

According to the different anesthesia techniques, they were divided into total intravenous anesthesia group (TIVA) and inhalational anesthesia group (INHA). In both groups, patients were induced anesthesia with midazolam 0.05 ~ 0.15 mg/kg, 0.5 ug/kg fentanyl, and 1 ~ 2.5 mg/kg propofol. In the TIVA group, anesthesia was maintained with propofol and remifentanil. In the INHA group, anesthesia was maintained with sevoflurane and remifentanil. Postoperative pain management was the same in both groups and neither has undergone epidural anesthesia.

### Indicator and data

The primary endpoint of this study is overall survival (OS), which was defined as the period from the patients’ date of surgery to the time of death, the secondary endpoint is disease-free survival (DFS), which was defined as the interval between the date of surgery and the date of tumor recurrence and metastasis or death. The date of last follow-up was reviewed for both endpoints until the death or follow-up deadline. The follow-up deadline was August 1, 2021. We collected the following perioperative information: demographic data, coexisting disease, adjuvant therapy (radiotherapy / chemotherapy), preoperative hemoglobin (HB), American Society of Anesthesiologists (ASA) grade, duration of anesthesia, the length of the operation, surgical type, degree of differentiation, pathological classification, tumor location, cancer staging, total hospital stay and postoperative hospital stay. The criteria for cancer staging are based on the 8th edition of the American Joint Committee on Cancer (AJCC) Cancer Staging Manual.

### Statistical analysis

Cases with substandard data were excluded from the final analysis, and cases meeting the requirements of this study were analyzed. Associations between categorical variables were assessed using the Fisher exact test or χ2 test. Continuous variables between patient groups were compared by T-tests or Manne Whitney U tests. Categorical data were represented by n (%), and analyzed by χ2 test, continuous data were expressed as the mean (standard deviation, SD) or median [interquartile range], and two independent samples were analyzed by T-tests. The OS and DFS were calculated by the Kaplan Meier method. Cox proportional hazards regression models were used to compare risk factors between the different groups by using univariate models. Cox proportional hazard regression model was used to compare the risk factors between different groups by single factor model. The significance variables and clinical significance variables in univariate analysis were analyzed by the multivariate analysis. Propensity score matching reduces between-group differences, choosing stabilized inverse probability of treatment weighting (SIPTW) for balance [10]. The propensity model included the following variables: sex, age, BMI, smoke, drink, hypertension, diabetes, cardiovascular disease, cerebrovascular disease, adjuvant treatment, preoperative HB, ASA, duration of anesthesia, the length of the operation, surgical type, degree of differentiation, blood transfusion, pathological classification, tumor location, T, N, TNM, total hospital stay, postoperative hospital stay. We used the package “survival”for the Cox regression analysis and the stabilized inverse probability of treatment weighting. All the analyses were performed by the R software (version 4.1.2). Forest plot was built by “forestplot”package and *P*-value < 0.05 was considered statistically significant.

## Results

**Fig. 1:**
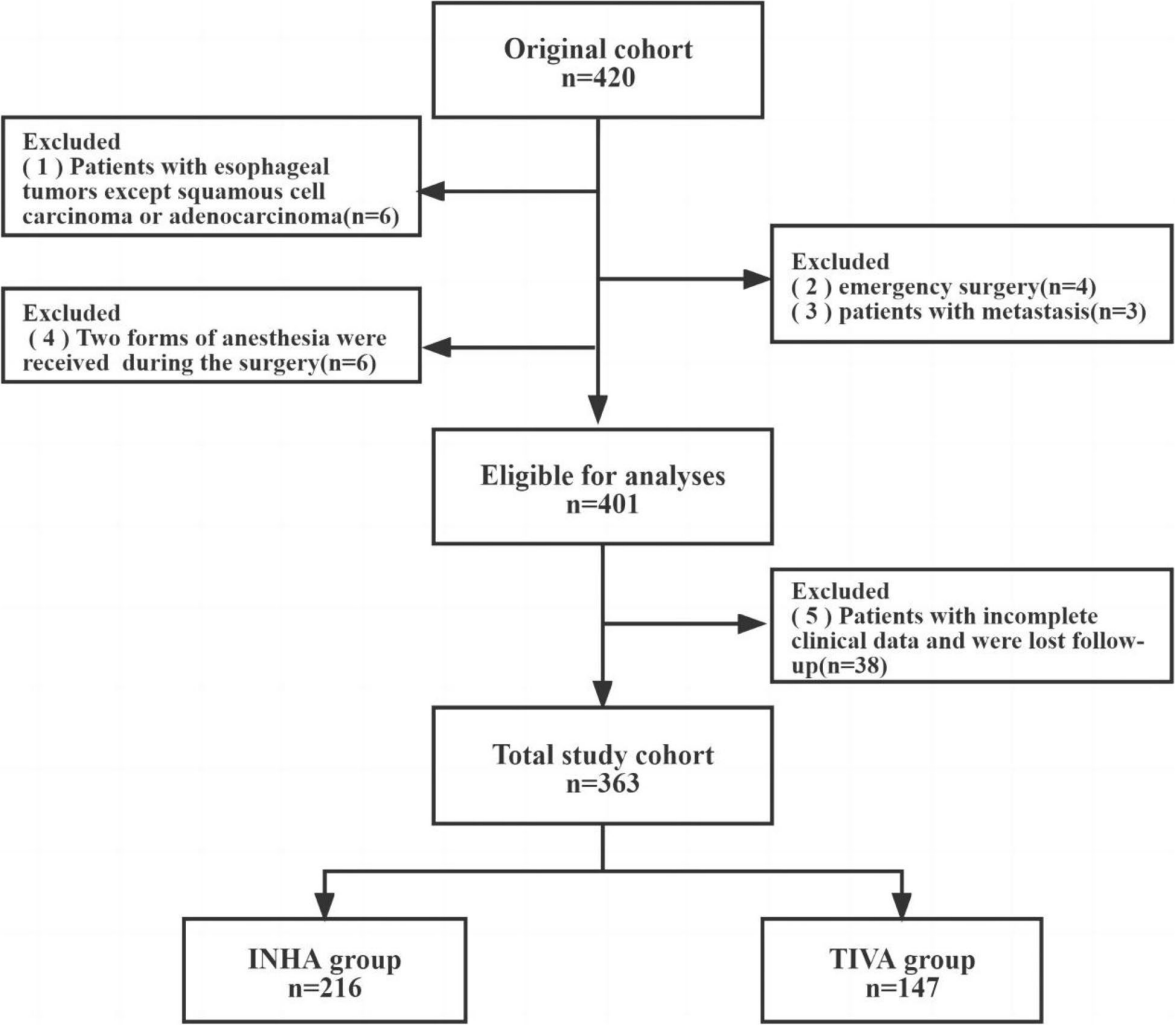
Inclusion and exclusion criteria for patients. INHA = Inhalational anesthesia; TIVA = Total intravenous anesthesia

All of the 420 patients underwent esophagectomy during the study period, and 57 patients met the exclusion criteria, a total of 363 patients entered the final analysis (INHA, *n* = 216, TIVA, *n* = 147, Fig. 1). Perioperative characteristics of patients in the entire cohort study group are shown in Table 1.

**Table 1:** Patient characteristics for before SIPTW adjustment and after SIPTW adjustment

^Variable^	^BEFORE SIPTW ADJUSTMENT^				^AFTER SIPTW ADJUSTMENT^			
^Group^	^INHA (*n*=216)^	^TIVA (*n*=147)^	^*P*^	^SMD^	^INHA (*n*=215.8)^	^TIVA (*n*=147.8)^	^*P*^	^SMD^
^Age^	^59^	^57^	^0.210^	^0.114^	^59^	^58^	^0.821^	^0.006^
^(Median [IQR] year)^	^[53.00, 63.25]^	^[53.00, 62.00]^			^[53.00, 63.00]^	^[53.00, 63.00]^		
^Adjuvant treatment (%)^			^0.292^	^0.124^			^0.897^	^0.015^
^ No^	^125 (57.9)^	^76 (51.7)^			^117.5 (54.4)^	^79.3 (53.7)^		
^ Yes^	^91 (42.1)^	^71 (48.3)^			^98.4 (45.6)^	^68.5 (46.3)^		
^ASA (%)^			^0.513^	^0.123^			^0.990^	^0.016^
^ I^	^38 (17.6)^	^21 (14.3)^			^35.3 (16.4)^	^23.3 (15.8)^		
^ Ii^	^158 (73.1)^	^108 (73.5)^			^158.5 (73.4)^	^109.5 (74.1)^		
^ Iii^	^20 ( 9.3)^	^18 (12.2)^			^22.0 (10.2)^	^15.0 (10.2)^		
^ BMI^	^22.1^	^22.5^	^0.717^	^0.076^	^22.1^	^22.1^	^0.525^	^0.021^
^ (Median [IQR] kg/m2)^	^[20.10, 24.83]^	^[20.30, 24.20]^			^[20.20, 24.99]^	^[20.22, 24.10]^		
^Blood transfusion (%)^			^0.310^	^0.145^			^0.774^	^0.039^
^ No^	^206 (95.4)^	^144 (98.0)^			^207.8 (96.3)^	^141.2 (95.5)^		
^ Yes^	^10 ( 4.6)^	^3 ( 2.0)^			^8.0 ( 3.7)^	^6.6 ( 4.5)^		
^Cardiovascular disease (%)^			^1.000^	^0.031^			^0.963^	^0.005^
^ No^	^209 (96.8)^	^143 (97.3)^			^209.3 (97.0)^	^143.5 (97.1)^		
^ Yes^	^7 ( 3.2)^	^4 ( 2.7)^			^6.5 ( 3.0)^	^4.3 ( 2.9)^		
^Cerebrovascular disease (%)^			^0.476^	^0.099^			^0.934^	^0.009^
^ No^	^205 (94.9)^	^136 (92.5)^			^203.3 (94.2)^	^138.9 (94.0)^		
^ Yes^	^11 ( 5.1)^	^11 ( 7.5)^			^12.5 ( 5.8)^	^8.9 ( 6.0)^		
^Drink (%)^			^0.401^	^0.103^			^0.880^	^0.018^
^ No^	^75 (34.7)^	^44 (29.9)^			^70.4 (32.6)^	^49.4 (33.4)^		
^ Yes^	^141 (65.3)^	^103 (70.1)^			^145.5 (67.4)^	^98.4 (66.6)^		
^Diabetes (%)^			^0.114^	^0.192^			^0.657^	^0.056^
^ No^	^212 (98.1)^	^139 (94.6)^			^205.7 (95.3)^	^142.5 (96.4)^		
^ Yes^	^4 ( 1.9)^	^8 ( 5.4)^			^10.1 ( 4.7)^	^5.3 ( 3.6)^		
^ Duration of anesthesia^	^6.1^	^6.3^	^0.189^	^0.178^	^6.23^	^6.2^	^0.886^	^0.002^
^ (Median [IQR] h)^	^[5.00, 7.00]^	^[5.20, 7.35]^			^[5.00, 7.00]^	^[5.00, 7.30]^		
^Degree of differentiation (%)^			^0.777^	^0.044^			^0.884^	^0.017^
^ Poor^	^165 (76.4)^	^115 (78.2)^			^165.4 (76.6)^	^114.3 (77.3)^		
^ Well^	^51 (23.6)^	^32 (21.8)^			^50.5 (23.4)^	^33.5 (22.7)^		
^Hypertension (%)^			^0.182^	^0.157^			^0.974^	^0.004^
^ No^	^186 (86.1)^	^118 (80.3)^			^181.0 (83.9)^	^124.2 (84.0)^		
^ Yes^	^30 (13.9)^	^29 (19.7)^			^34.8 (16.1)^	^23.7 (16.0)^		
^N (%)^			^0.009^	^0.329^			^0.985^	^0.020^
^ N0^	^117 (54.2)^	^61 (41.5)^			^103.7 (48.0)^	^70.6 (47.8)^		
^ N1^	^58 (26.9)^	^62 (42.2)^			^72.2 (33.4)^	^48.7 (32.9)^		
^ N2^	^41 (19.0)^	^24 (16.3)^			^40.0 (18.5)^	^28.5 (19.3)^		
^ Postoperative hospital stay^	^16^	^15^	^0.906^	^0.038^	^16^	^15^	^0.921^	^0.010^
^ (Median [IQR] d)^	^[14.00, 18.25]^	^[14.00, 18.50]^			^[14.00, 18.00]^	^[14.00, 18.38]^		
^ Preoperative HB^	^146.5^	^145.1^	^0.909^	^0.035^	^146.64^	^145^	^0.761^	^0.023^
^ (Median [IQR] g/l)^	^[135.93, 154.00]^	^[137.00, 154.40]^			^[136.23, 154.21]^	^[136.44, 154.76]^		
^Pathological classification (%)^			^0.590^	^0.100^			^0.636^	^0.051^
^ Adenocarcinoma^	^6 ( 2.8)^	^2 ( 1.4)^			^4.5 ( 2.1)^	^2.1 ( 1.4)^		
^ Squamous cell carcinoma^	^210 (97.2)^	^145 (98.6)^			^211.3 (97.9)^	^145.7 (98.6)^		
^Sex (%)^			^1.000^	^0.003^			^0.931^	^0.009^
^ Female^	^6 ( 2.8)^	^4 ( 2.7)^			^5.3 ( 2.4)^	^3.4 ( 2.3)^		
^ Male^	^210 (97.2)^	^143 (97.3)^			^210.6 (97.6)^	^144.4 (97.7)^		
^Smoke (%)^			^0.333^	^0.116^			^0.919^	^0.012^
^ No^	^84 (38.9)^	^49 (33.3)^			^78.0 (36.1)^	^52.6 (35.6)^		
^ Yes^	^132 (61.1)^	^98 (66.7)^			^137.8 (63.9)^	^95.2 (64.4)^		
^ The length of the operation^	^5.2^	^5.3^	^0.160^	^0.194^	^5.2^	^5.11^	^0.756^	^0.004^
^ (Median [IQR] h)^	^[4.07, 6.10]^	^[3.95, 6.40]^			^[4.10, 6.20]^	^[3.80, 6.17]^		
^Surgical type (%)^			^0.835^	^0.095^			^0.988^	^0.039^
^ Lvor lewis^	^86 (39.8)^	^58 (39.5)^			^83.6 (38.7)^	^54.8 (37.1)^		
^ Mckeown^	^55 (25.5)^	^37 (25.2)^			^54.7 (25.3)^	^38.4 (26.0)^		
^ Sweet^	^74 (34.3)^	^50 (34.0)^			^76.3 (35.4)^	^53.5 (36.2)^		
^ Thoracoabdominal incision^	^1 ( 0.5)^	^2 ( 1.4)^			^1.2 ( 0.6)^	^1.1 ( 0.7)^		
^Tumor location (%)^			^0.739^	^0.083^			^0.956^	^0.035^
^ Lower esophagus^	^96 (44.4)^	^67 (45.6)^			^96.5 (44.7)^	^64.0 (43.3)^		
^ Middle esophagus^	^105 (48.6)^	^67 (45.6)^			^100.7 (46.6)^	^69.9 (47.3)^		
^ Upper esophagus^	^15 ( 6.9)^	^13 ( 8.8)^			^18.7 ( 8.6)^	^14.0 ( 9.4)^		
^T (%)^			^0.884^	^0.086^			^0.986^	^0.040^
^ T1^	^51 (23.6)^	^32 (21.8)^			^48.6 (22.5)^	^34.6 (23.4)^		
^ T2^	^40 (18.5)^	^32 (21.8)^			^44.1 (20.4)^	^31.7 (21.4)^		
^ T3^	^121 (56.0)^	^80 (54.4)^			^119.5 (55.4)^	^79.4 (53.7)^		
^ T4^	^4 ( 1.9)^	^3 ( 2.0)^			^3.6 ( 1.7)^	^2.1 ( 1.5)^		
^TNM (%)^			^0.261^	^0.177^			^0.999^	^0.004^
^ I^	^46 (21.3)^	^23 (15.6)^			^39.9 (18.5)^	^27.5 (18.6)^		
^ Ii^	^75 (34.7)^	^48 (32.7)^			^72.0 (33.4)^	^49.2 (33.3)^		
^ Iii^	^95 (44.0)^	^76 (51.7)^			^104.0 (48.2)^	^71.1 (48.1)^		
^ Total hospital stay^	^24^	^24^	^0.627^	^0.029^	^24^	^24^	^0.567^	^0.017^
^ (Median [IQR] d)^	^[21.00, 28.00]^	^[21.00, 28.50]^			^[21.00, 27.04]^	^[21.00, 29.00]^		

Abbreviations: *IQR* Inter-quartile range, *ASA* American society of anesthesiologists, *BMI* Body mass index, *INHA* Inhalational anesthesia, *TIVA* Total intravenous anesthesia, *SIPTW* Stabilized inverse probability of treatment weighting, *SMD* Standardized mean difference

**Fig. 2:**
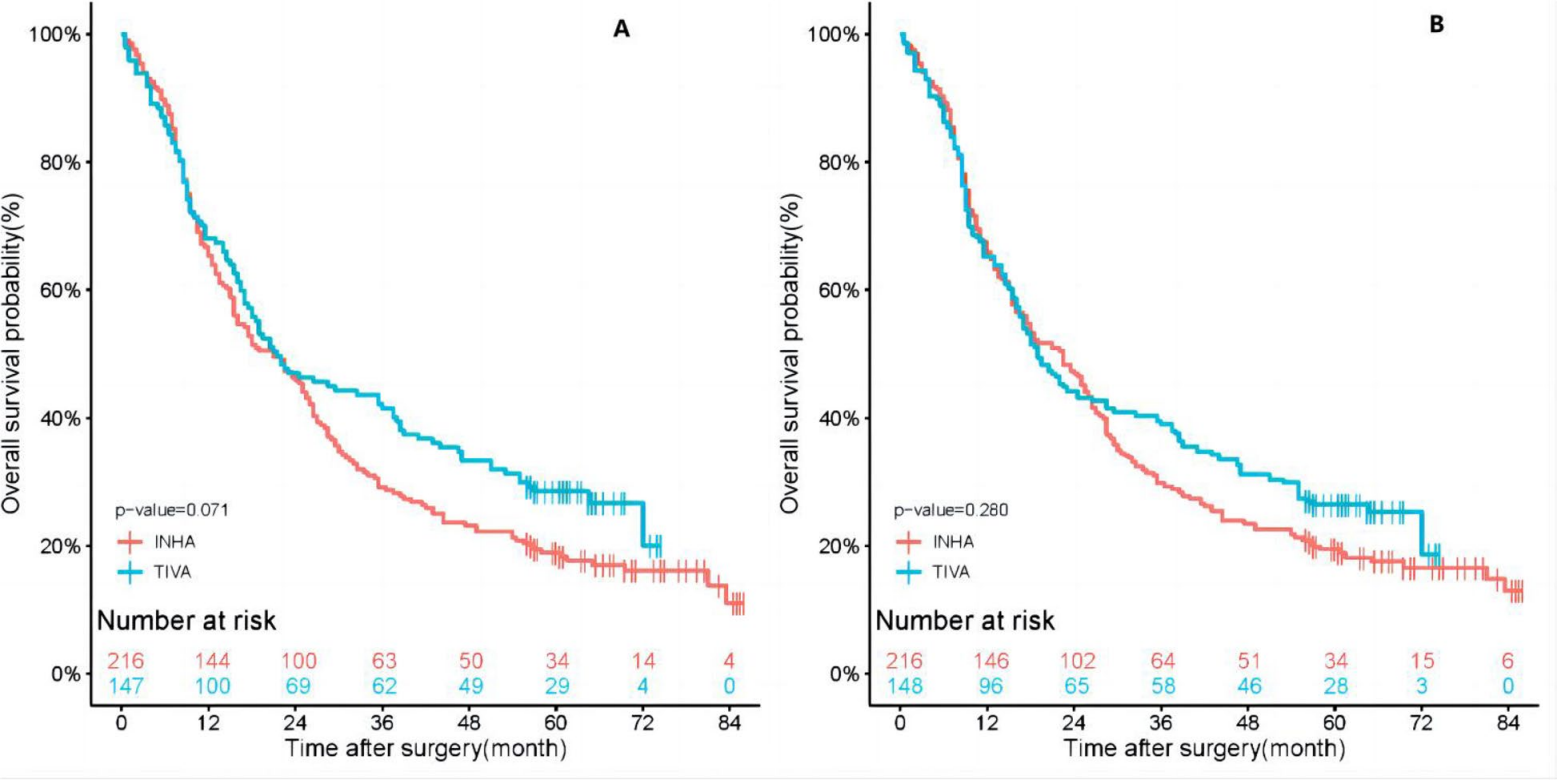
**A** Kaplan–Meier survival curve for overall survival before SIPTW. **B** Kaplan–Meier survival curve for overall survival after SIPTW. SIPTW stabilized inverse probability of treatment weighting

**Fig. 3:**
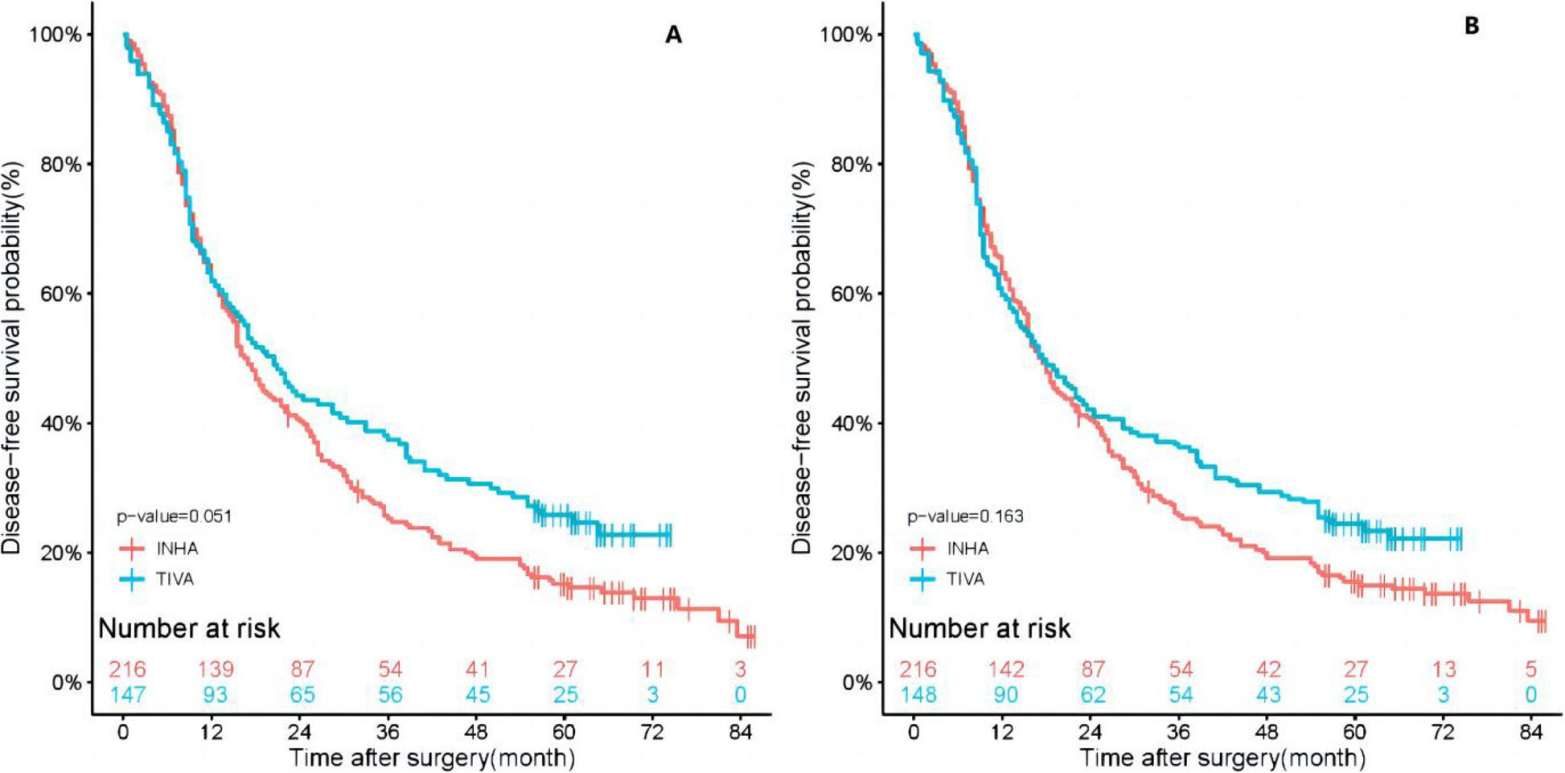
**A** Kaplan–Meier survival curve for disease-free survival before SIPTW. **B** Kaplan–Meier survival curve for disease-free survival after SIPTW. SIPTW stabilized inverse probability of treatment weighting

In this study, the median follow-up time for all patients was 21.00 months (interquartile range 9.00 to 54.75 months), 21.50 months in follow-up in TIVA group (interquartile range 9.00 to 57.00 months), and 21.00 months in INHA group (interquartile range 9.38 to 43.38 months). The results of the Kaplan–Meier survival curves are shown in Fig. 2, the 1-year and 3-year OS rates for TIVA group were 68.0% and 41.5%. The 1-year and 3-year OS rates of INHA patients were 65.3% and 29.2%. After SIPTW, the 1-year and 3-year OS rates for TIVA patients were 65.2% and 39.0%, in INHA group were 65.9% and 29.8%. And in Fig. 3, the 1-year and 3-year DFS rates of TIVA patients were 61.9% and 37.4%. The 1-year and 3-year DFS rates of INHA group were, respectively, 62.0% and 25.2%. After SIPTW, the 1-year and 3-year DFS rates of TIVA patients were 59.8% and 36.3%, and in INHA group were 63.1% and 25.7%. The results showed that there were no significant differences in overall survival (*P* = 0.280) or disease-free survival (*P* = 0.163) between the TIVA and INHA groups.

In the SIPTW cohort, we established Cox proportional hazard models for overall survival and disease-free survival to assess the relationship between different types of anesthesia and overall survival or disease-free survival. Univariate Cox regression revealed no significant association between TIVA and poorer OS (HR, 0.87, 95%CI, 0.67 to 1.13, *P* = 0.307) or DFS (HR, 0.84, 95%CI, 0.65 to 1.09, *P* = 0.192) when compared with the INHA group (Tables 2 and 3).

**Table 2:** Univariate analysis of OS

		^SIPTW−OS^	
^Variable^	^HR^	^95%CI^	^*P*^
^ASA II^	^1.46^	^1.00–2.13^	^0.052^
^ASA III^	^2.35^	^1.26–4.36^	^0.007^
^Adjuvant treatment Yes^	^0.76^	^0.59–0.97^	^0.028^
^Blood transfusion Yes^	^1.09^	^0.63–1.88^	^0.755^
^Duration of anesthesia^	^1.01^	^0.94–1.09^	^0.717^
^Degree of differentiation^	^0.74^	^0.55–0.98^	^0.039^
^Group^	^0.87^	^0.67–1.13^	^0.307^
^Postoperative hospital stay^	^1.01^	^1.00–1.02^	^0.199^
^Pathological classification^ ^Squamous carcinoma^	^2.03^	^0.58–7.13^	^0.269^
^Surgical type McKeown^	^1.21^	^0.92–1.59^	^0.179^
^Surgical type Sweet^	^0.80^	^1.26–0.58^	^0.145^
^Surgical type Thoracoabdominal incision^	^1.04^	^0.96–0.64^	^0.865^
^TNM II^	^1.17^	^0.80–1.70^	^0.410^
^TNM III^	^1.57^	^1.10–2.23^	^0.013^
^Tumor location Middle esophagus^	^1.28^	^0.98–1.66^	^0.072^
^Tumor location Upper esophagus^	^1.54^	^1.04–2.29^	^0.031^
^Total hospital stay^	^1.01^	^1.00–1.03^	^0.044^
^The length of the operation^	^1.01^	^0.94–1.10^	^0.727^

**Table 3:** Univariate analysis of DFS

		^SIPTW−DFS^	
^Variable^	^HR^	^95%CI^	^*P*^
^ASA II^	^1.47^	^1.02–2.12^	^0.041^
^ASA III^	^2.72^	^1.53–4.86^	^0.001^
^Adjuvant treatment Yes^	^0.95^	^0.74–1.21^	^0.660^
^Blood transfusion Yes^	^1.01^	^0.58–1.75^	^0.983^
^Duration of anesthesia^	^1.02^	^0.95–1.10^	^0.553^
^Degree of differentiation^	^0.75^	^0.57–0.99^	^0.044^
^Group^	^0.84^	^0.65–1.09^	^0.192^
^Postoperative hospital stay^	^1.01^	^1.00–1.03^	^0.106^
^Pathological classification^ ^Squamous carcinoma^	^1.73^	^0.57–5.24^	^0.330^
^Surgical type McKeown^	^1.20^	^0.92–1.58^	^0.186^
^Surgical type Sweet^	^0.74^	^0.55–1.01^	^0.056^
^Surgical type Thoracoabdominal incision^	^0.91^	^0.57–1.45^	^0.688^
^TNM II^	^1.23^	^0.84–1.80^	^0.283^
^TNM III^	^1.67^	^1.17–2.39^	^0.005^
^Tumor location middle esophagus^	^1.23^	^0.95–1.59^	^0.124^
^Tumor location upper esophagus^	^1.29^	^0.86–1.94^	^0.223^
^Total hospital stay^	^1.02^	^1.00–1.03^	^0.013^
^The length of the operation^	^1.02^	^0.95–1.10^	^0.558^

**Fig. 4:**
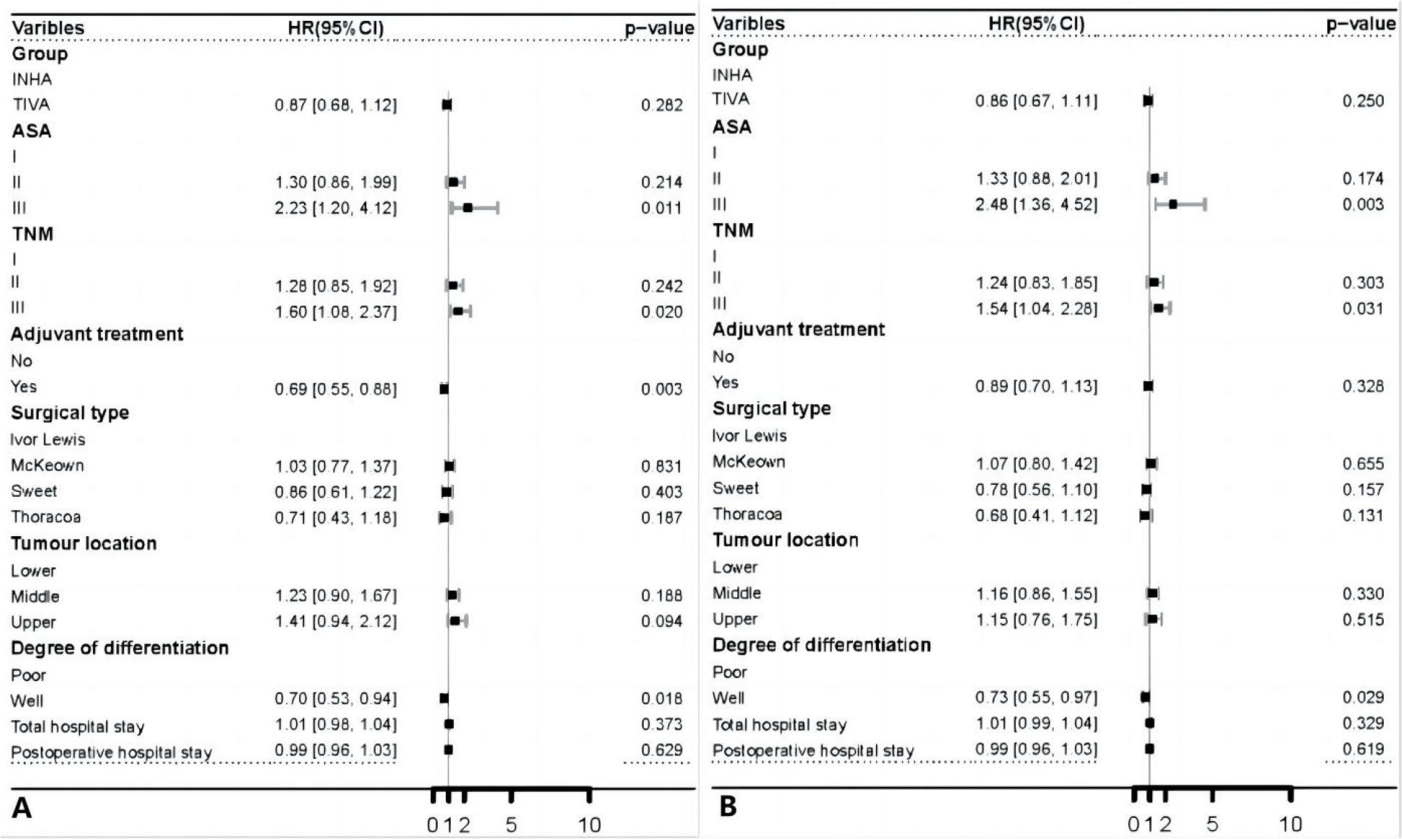
**A** Forest plot for multivariable cox proportional of overall survival after SIPTW. **B** Forest plot for multivariable cox proportional of disease-free survival after SIPTW

In the multivariate cox model, factors with *P* < 0.1 in univariate cox regression or clinically important factors were considered. The results showed that TIVA and IHNA had no significant effect of improving OS (HR, 0.87, 95%CI, 0.68 to 1.12,* P* = 0.282) or DFS (HR, 0.86, 95%CI, 0.67 to 1.11,* P* = 0.250) in patients undergoing esophageal cancer surgery. The OS and DFS were also confirmed as worse for ASA III (HR, 2.23, 95%CI, 1.20 to 4.12,* P* = 0.011, HR, 2.48, 95%CI, 1.36 to 4.52, *P* = 0.003) and TNM III (HR, 1.60, 95%CI, 1.08 to 2.37,* P* = 0.020, HR, 1.54, 95%CI, 1.04 to 2.28, *P* = 0.031). The adjuvant therapy (HR, 0.69, 95%CI, 0.55 to 0.88, *P* = 0.003) was statistically significant in improving OS. Degree of differentiation (HR, 0.70, 95%CI, 0.53 to 0.94, *P* = 0.018, HR, 0.73, 95%CI, 0.55 to 0.97, *P* = 0.029) was correlated with OS and DFS (Fig. 4).

## Discussion

This study showed that there was no significant correlation between total intravenous anesthesia and inhalational anesthesia for overall survival (OS) in patients undergoing esophagectomy, TIVA did not improve the disease-free survival (DFS) of patients. Our results were inconsistent with the results of a retrospective study, which found that intravenous anesthesia with propofol during esophageal cancer surgery was related to better postoperative survival rates compared with volatile anesthesia [2].

Whether different anesthetics can affect the prognosis of tumor patients is still controversial. In a retrospective study of the overall survival of pancreatic cancer patients with different anesthesia methods showed TIVA and IHNA for improved OS in pancreatic patients or DFS was not significant [11]. Another study showed that TIVA was not significantly associated with a reduction in overall 1-year mortality or cancer-related mortality after gastric cancer surgery compared to IHNA [12]. There were no association between the type of general anesthesia used in cancer surgery and the overall, 1-year, and 5-year survival rates after surgery [13].

The immunomodulatory effect of anesthetics is considered to be an important mechanism for the effect of anesthetics on tumor prognosis [7]. Sevoflurane has been shown to induce T-lymphocyte apoptosis, reduce natural killer (NK) cell activity, reduce Th1 / Th2 ratio, increase tumorigenic cytokines and matrix metalloproteinases (MMPS) levels (key enzymes involved in basement membrane disassembly, thereby promoting tumor dissemination) to systematically damage immune function [14, 15]. In contrast, propofol increased the activity of cytotoxic t-lymphocytes, does not inhibit the activity of the NK cells, and reduced the tumorigenic cytokines, has the protective and anticancer effects [16]. Propofol can also inhibit matrix metalloproteinases, it has the anti-tumor characteristics [7].

Perioperative variables such as intraoperative blood transfusion, degree of differentiation, and ASA grade may affect the immunomodulation, thus leading to recurrence or metastasis after cancer surgery. Blood transfusions can carry the risk of spreading disease, also have been shown to be associated with poor prognosis recently [17]. A retrospective cohort study of perioperative transfusion on overall survival after esophageal squamous cell carcinoma (ESCC) concluded that perioperative transfusion had no effect on the overall survival of ESCC patients [18]. Our study also confirmed that OS in esophageal cancer patients was not associated with transfusion, and DFS was not associated with transfusion, which was consistent with previous studies. In the multivariate COX risk proportional regression model, the degree of differentiation was associated with OS, which was consistent with the findings of previous studies: a retrospective study of the clinical characteristics and prognostic factors for the surgical treatment of elderly esophageal squamous cell carcinoma showed the degree of differentiation of tumor cells was significantly higher for prognostic factors and survival rates in the higher differentiated group than in the middle and lower differentiated groups [19]. ASA grade is used preoperative to assess patient status and patient status was associated with metastasis and recurrence [20]. In our study, the OS and DFS were also confirmed as worse for ASA III than for ASA I.

Our study also collected primary tumor location, univariate analysis showed that after SIPTW(Table 2), patients with tumor location in the upper esophagus had a higher risk of death than those in the lower esophagus. This may be because the upper esophagus includes the neck and upper thoracic segments. Patients with proximal esophageal cancer often present with locally advanced disease, potentially curative surgery requiring extensive cutting resection, the risk of postoperative complications is higher [21]. However, in the multivariate analysis, the result was negative, which may be due to the number of patients with upper esophageal cancer in the data we collected was too small. The upper esophageal cancer tumors are relatively rare, and only accounts for 10% of all esophageal cancer cases [22].

### Limitations

Firstly, this is a retrospective, single-center, observational study, and the sample size of this study was small, a larger sample size is needed, multi-center research data are even more convincing. Secondly, we performed a multivariate analysis and a propensity-matched analysis of the variables, but we could not rule out some unmeasured confounding factors that might affect the results. Thirdly, the tumor subtypes included in this study were esophageal squamous cell carcinoma and esophageal adenocarcinoma, no other uncommon subtypes.

## Conclusion

Intravenous anesthesia and inhalational anesthesia have no effect on the overall survival and disease-free survival for patients undergoing esophageal cancer surgery.

## Data Availability

All data generated or analyzed during this study are included in this published article.

## Abbreviations

TIVA: Total intravenous anesthesia
INHA: Inhalational anesthesia
SIPTW: Stabilized inverse probability of treatment weighting
OS: Overall survival
DFS: Disease-free survival
ASA: American society of anesthesiologists
AJCC: American joint committee on cancer
NK: Natural killer
